# Supplementary material for: Lung Tumor Cells with Different Tn Antigen Expression Present Distinctive Immunomodulatory Properties
Source: Int J Mol Sci. 2022 Oct 10;23(19):12047. doi: 10.3390/ijms231912047 (PMC9570357; doi:10.3390/ijms231912047)
Supplement: Supplementary file 1 [file ijms-23-12047-s001.zip › ijms-1875374-supplementary.pdf]

Supplementary Material

The Tn antigen confers distinctive immunomodulatory properties to lung tumor cells

Table S1. Plant lectin primary and secondary specificity to different glycan structures.

Lectin	Abbreviation	Primary specificity	Secondary specificity	Reference
Vicia villosa isolectine B4	**VVL**	Tn, α-GalNAc	β-GalNAc	[51]
Jacalin	**JAC**	Galβ(1-3)GalNAc, α-GalNAc	*Core* 3, sialyl-T	[52]
Helix pomatia agglutinin	**HPA**	α/ β-GalNAc		[53]
Glycine max agglutinin	**SBA**	α/β-GalNAc, Tn	Gal	[54]
Dolichos biflorus agglutinin	**DBA**	α-GalNAc, blood group A		[55]
Maackia amurensis II agglutinin	**MAL II**	NeuAcα(2-3)Gal		[56]
Sambucus Nigra agglutinin	**SNA**	NeuAcα(2-6)Gal	NeuAcα(2-6)GalNAc, Lac	[57]

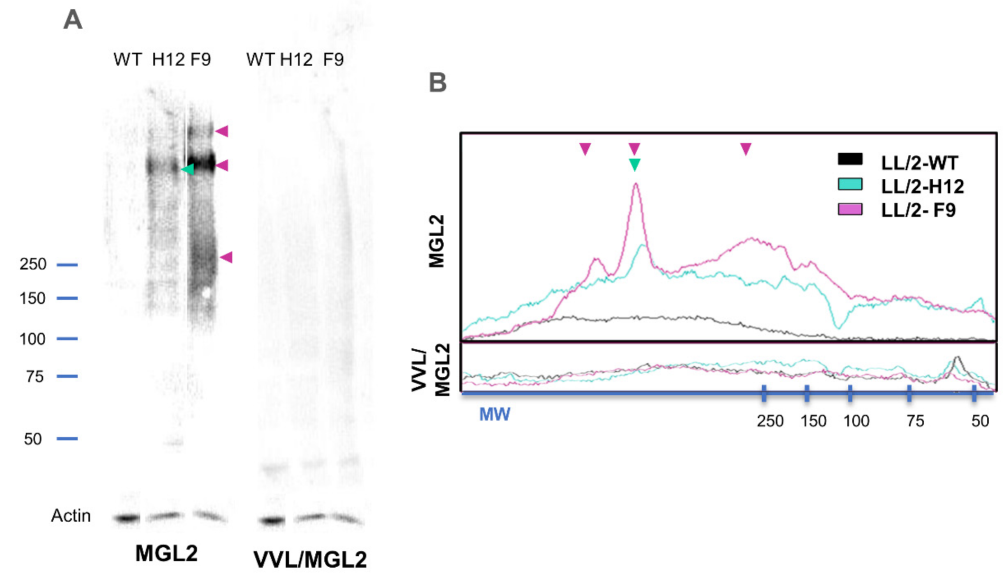

Figure S1. VVL lectin inhibits MGL2 recognition of both LL/2 Tn⁺ cells. (**A,B**) MGL2 recognition of whole cell lysates by Western blotting. Membranes were pre-incubated with VVL and developed with MGL2-Fc Green and violet arrows represent components recognized in LL/2-H12 and LL/2-F9 cells respectively. (**B**) Densitometry of Western blot bands after MGL2-Fc recognition, with or without pre-incubation with VVL.

Supplementary References

51. Puri, K.D.; Gopalakrishnan, B.; Surolia, A. Carbohydrate binding specificity of the Tn-antigen binding lectin fromVicia villosaseeds (VVLB4). *FEBS Lett.* **1992**, *312*, 208–212.
52. Hagiwara, K.; Collet-Cassart, D.; Kobayashi, K.; Vaerman, J.P. Jacalin: isolation, characterization, and influence of various factors on its interaction with human IgA1, as assessed by precipitation and latex agglutination. *Mol. Immunol.* **1988**, *25*, 69–83.
53. Sanchez, J.-F.; Lescar, J.; Chazalet, V.; Audfray, A.; Gagnon, J.; Alvarez, R.; Breton, C.; Imberty, A.; Mitchell, E.P., Biochemical and Structural Analysis of Helix pomatia Agglutinin. *J. Biol. Chem.* **2006**, *281*, 20171–20180.
54. Dam, T.K.; Gerken, T.A.; Cavada, B.S.; Nascimento, K.S.; Moura, T.R.; Brewer, C.F. Binding Studies of α-GalNAc-specific Lectins to the α-GalNAc (Tn-antigen) Form of Porcine Submaxillary Mucin and Its Smaller Fragments. *J. Biol. Chem.* **2007**, *282*, 28256–28263.
55. Piller, V.; Piller, F.; Cartron, J.-P. Comparison of the carbohydrate-binding specificities of seven N-acetyl-D-galactosamine-recognizing lectins. *Eur. J. Biochem.* **1990**, *191*, 461–466.
56. Geisler, C.; DJarvis, L. Letter to the Glyco-Forum: Effective glycoanalysis with Maackia amurensis lectins requires a clear understanding of their binding specificities. *Glycobiology* **2011**, *21*, 988–993.
57. Shibuya, N.; Goldstein, I.J.; Broekaert, W.F.; Nsimba-Lubaki, M.; Peeters, B.; Peumans, W.J. The elderberry (*Sambucus nigra* L.) bark lectin recognizes the Neu5Ac(alpha 2-6)Gal/GalNAc sequence. *J. Biol. Chem.* **1987**, *262*, 1596–1601.
